# Supplementary material for: MetaRibo-Seq measures translation in microbiomes
Source: Nat Commun. 2020 Jun 29;11:3268. doi: 10.1038/s41467-020-17081-z (PMC7324362; doi:10.1038/s41467-020-17081-z)
Supplement: Supplementary file 10 — Supplementary Data 7 [file 41467_2020_17081_MOESM10_ESM.zip › File2/Confidence_High_Taxonomy/159093_out.krona.html]

Javascript must be enabled to view this page.

members
magnitude
magnitudeUnassigned
count
unassigned
taxon
rank

159093\_out

7

2
7
superkingdom

976
7
phylum

200643
7
class

171549
7
order

family
171550
7

7
239759
genus

species
6
328814

SRS015960\_contig\_number\_contig-100\_1617.137924SRS022071\_contig\_number\_30073SRS052697\_contig\_number\_42796SRS053398\_contig\_number\_11541SRS1055034\_contig\_number\_contig-100\_1732.79795SRS1055043\_contig\_number\_13759

1
1896969
species

SRS015217\_contig\_number\_contig-100\_1722.179701
